# Construction of a highly efficient CRISPR/Cas9-mediated duck enteritis virus-based vaccine against H5N1 avian influenza virus and duck Tembusu virus infection

**DOI:** 10.1038/s41598-017-01554-1

**Published:** 2017-05-03

**Authors:** Zhong Zou, Kun Huang, Yanmin Wei, Huanchun Chen, Ziduo Liu, Meilin Jin

**Affiliations:** 1 State Key Laboratory of Agricultural Microbiology, Huazhong Agricultural University, Wuhan, 430070 P.R. China; 2 College of Veterinary Medicine, Huazhong Agricultural University, Wuhan, 430070 P.R. China; 3 College of Life Sciences, Huazhong Agricultural University, Wuhan, 430070 P.R. China; 4 Key Laboratory of development of veterinary diagnostic products, Ministry of Agriculture, Wuhan, Hubei People’s Republic of China

## Abstract

Duck enteritis virus (DEV), duck tembusu virus (DTMUV), and highly pathogenic avian influenza virus (HPAIV) H5N1 are the most important viral pathogens in ducks, as they cause significant economic losses in the duck industry. Development of a novel vaccine simultaneously effective against these three viruses is the most economical method for reducing losses. In the present study, by utilizing a clustered regularly interspaced short palindromic repeats (CRISPR)/associated 9 (Cas9)-mediated gene editing strategy, we efficiently generated DEV recombinants (C-KCE-HA/PrM-E) that simultaneously encode the hemagglutinin (HA) gene of HPAIV H5N1 and pre-membrane proteins (PrM), as well as the envelope glycoprotein (E) gene of DTMUV, and its potential as a trivalent vaccine was also evaluated. Ducks immunized with C-KCE-HA/PrM-E enhanced both humoral and cell-mediated immune responses to H5N1 and DTMUV. Importantly, a single-dose of C-KCE-HA/PrM-E conferred solid protection against virulent H5N1, DTMUV, and DEV challenges. In conclusion, these results demonstrated for the first time that the CRISPR/Cas9 system can be applied for modification of the DEV genome rapidly and efficiently, and that recombinant C-KCE-HA/PrM-E can serve as a potential candidate trivalent vaccine to prevent H5N1, DTMUV, and DEV infections in ducks.

## Introduction

Waterfowls are naturally susceptible to several kinds of pathogens, such as highly pathogenic avian influenza virus (HPAIV) H5N1, duck Tembusu virus (DTMUV), and duck enteritis virus (DEV)^1–3^. HPAIV H5N1 has been documented in more than 60 countries. Since its first discovery in geese in Guangdong Province^4^, HPAIV H5N1 has resulted in frequent outbreaks in domestic poultry farms in China, and has resulted in millions of deaths of poultry animals. More importantly, H5N1 has overcome the species barrier and has infected humans^5^. Human infection usually occurs through direct exposure to infected poultry and wild birds, and as of the 3^rd^ October, 2016, there have been 856 confirmed human cases of H5N1 virus infection and 452 deaths (http://www.who.int/influenza/human_animal_interface/2016_10_03_tableH5N1.pdf), with fatality rates approaching 53%. Thus, HPAIV H5N1 has resulted in devastating damage to the poultry industry and also represents a severe threat to human health.

DTMUV is a newly identified flavivirus which has rapidly circulated among major duck-producing regions in China, Malaysia, and Thailand since April, 2010^6, 7^. Infected short-lived meat ducks display loss of appetite, retarded growth, and neurological symptoms, whereas egg-laying ducks exhibit dramatic reduction in egg production by 20–60%^8^. Moreover, evidence has shown that geese, chickens, and sparrows can be infected with this virus^9^. Therefore, the emergence of DTMUV has resulted in additional burden on the poultry industry. Additionally, serum samples of duck industry workers have shown high levels of antibody against DTMUV^10^, so the potential threats of DTMUV to public health are also high.

DEV, also called “duck plague”, is an acute, contagious, and lethal disease affecting ducks, swans, and geese. The DEV genome consists of approximately 160 kilobase pairs (kbp), each pair is composed of two unique sequences, unique long (UL) and unique short (US). The latter is flanked by inverted repeated sequences (IRS and TRS)^11^. DEV is a major cause of duck viral enteritis disease, which has a high mortality rate^3^. Obviously, the development of a trivalent vaccine that simultaneously acts against HPAIV H5N1, DTMUV, and DEV is the most economical method for reducing losses in the poultry industry. The DEV C-KCE strain, attenuated in the embryonated chicken egg strain, has routinely been used as live vaccine in ducks for over 50 years without concerns for human or animal safety. Recombinant avirulent DEV is currently being explored as a candidate vaccine carrier for multiple pathogens, both in ducks and chickens^12–15^. Several advantages associated with DEV vectors make them highly attractive as viral vector platforms, including their safety profile, ease of generation, ability to induce broad and strong immune responses, safety profile, and the ability to differentiate between infected and vaccinated animals^13^.

Several approaches have been developed to edit the DEV genome^12, 16, 17^. The current available method for modification of DEV is based on homologous recombination, bacterial artificial chromosome, and fosmid system construction. However, these traditional approaches for generating recombinant DEV are often inefficient, time-consuming, and labor-intensive due to the requirement of several rounds of plaque purification and transfer vector cloning procedures. Thus, a more efficient and straightforward genome editing technology for constructing recombinant DEV is needed.

Type II bacterial clustered regularly interspaced short palindromic repeats (CRISPR)/associated 9 (Cas9) is part of the bacterial acquired immune system against invading viruses^18^. CRISPR/Cas9-mediated genome editing technology has been successfully used for genetic engineering, including the generation of transgenic animals and knock-out or knock-in cell lines^19, 20^. Recently, proof-of-concept studies have shown that CRISPR/Cas9 has also been applied for editing the genomes of a number of large DNA viruses including adenovirus, I herpes simplex virus, Epstein-Barr virus, Pseudorabies virus, cytomegaloviruses, and vaccinia virus^21–27^. However, this technology has not been evaluated in DEV.

In the present study, we took advantage of the properties of C-KCE to generate a potential trivalent vaccine simultaneously harboring the hemagglutinin (HA) gene of HPAIV H5N1, pre-membrane proteins (PrM), and envelope glycoprotein (E) gene of DTMUV based on the CRISPR/Cas9-mediated gene editing strategy. The recombinant viruses (C-KCE-HA/PrM-E) were further evaluated *in vitro* and *in vivo* for levels of protein expression, stability, safety, and protection efficacy against HPAIV H5N1, DTMUV, and DEV challenge in ducks.

## Results

### Rapid generation of recombinant virus C-KCE-HA/PrM-E encoding HA and PrM-E based on CRISPR/Cas9 mediated gene editing

**Figure 1:**
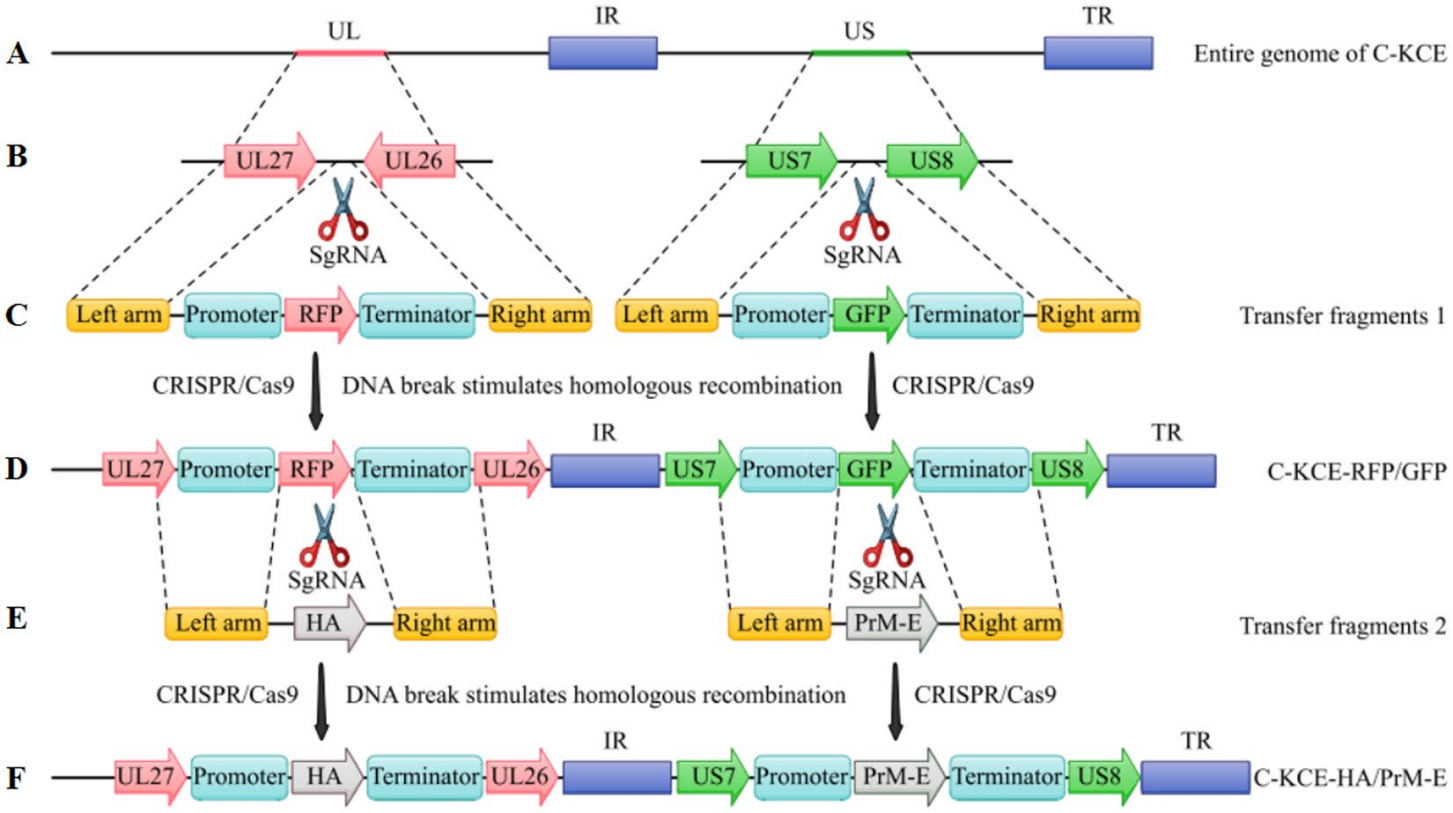
Schematic illustration of the novel candidate trivalent vaccine C-KCE-HA/PrM-E development. (**A**) Full-length of the attenuated commercial DEV vaccine strain (C-KCE). (**B**) Two portions of the genome C-KCE expanded to show the UL27, UL26, US7, and US8, and the gene junction regions are depicted. (**C**) The organization of transfer fragments Left arm-RFP-Right arm and Left arm-GFP-Right arm. (**D**) The recombinant C-KCE-RFP/GFP was generated using CRISPR/Cas9 system. (**E**) The organization of transfer fragments Left arm-HA-Right arm and Left arm-PrM-E-Right arm. (**F**) Following the CRISPR/Cas9 system mediated recombination, the recombinant C-KCE-HA/PrM-E was generated.

To exclude the adverse effects of viral gene modification on viral replication, the two gene junctions UL27/UL26 and US7/US8 of C-KCE, which our recent studies have proven to be suitable for foreign gene insertion^13, 28^, were selected as the target regions for recombinant C-KCE generation (Fig. 1A and B).

Two individual single guide RNAs (sgRNAs) targeting the two gene junctions UL27/UL26 and US7/US8 were cloned into pX330 in the gRNA cloning vector, and designated as gRNA-1 and gRNA-2, respectively. One shuttle fragment Left arm-red fluorescent protein (RFP)-Right arm carrying RFP for the gene junction between UL27 and UL26, and another donor fragment Left arm-green fluorescent protein (GFP)-Right arm containing GFP for the gene junction between US7 and US8 (Fig. 1C), were used for homologous recombination. To improve the efficiency of homology-directed repair (HDR), the error-prone non-homologous end joining (NHEJ) inhibitor SCR7 was introduced to the CRISPR/Cas9 system. Moreover, the single cell fluorescence-activated cell sorting (FACS) technique was also applied to facilitate plaque purification. Through CRISPR/Cas9 mediated recombination, the single cells positive for both RFP and GFP signals were sorted into a 24 well plate pre-seeded with chicken embryo fibroblast (CEF) cells. The progeny virus positive for both RFP and GFP were then purified with three rounds of plaque. We designated the purified recombinant C-KCE virus as C-KCE-RFP/GFP (Fig. 1D). This result suggests that the CRISPR/Cas9 system can simultaneously and efficiently mediate homologous recombination across two target sites in the C-KCE genome.

Given this result, we applied the CRISPR/Cas9 technique to generate a trivalent vaccine based on C-KCE. To accomplish this, two individual sgRNAs targeting RFP and GFP were cloned into the gRNA cloning vector pX330, and named as gRNA-3 and gRNA-4, respectively. HA derived from HPAIV H5N1 and PrM-E derived from DTMUV are excellent targets for vaccine development. One shuttle fragment Left arm-HA-Right arm carrying HA and another donor fragment Left arm-PrM-E-Right arm containing PrM-E were used for homologous recombination (Fig. 1E). The RFP and GFP regions were entirely replaced by HA and PrM-E (Fig. 1F), respectively. Thus, plaques that lacked double fluorescence were obtained through approximately three rounds of plaque purification.

**Figure 2:**
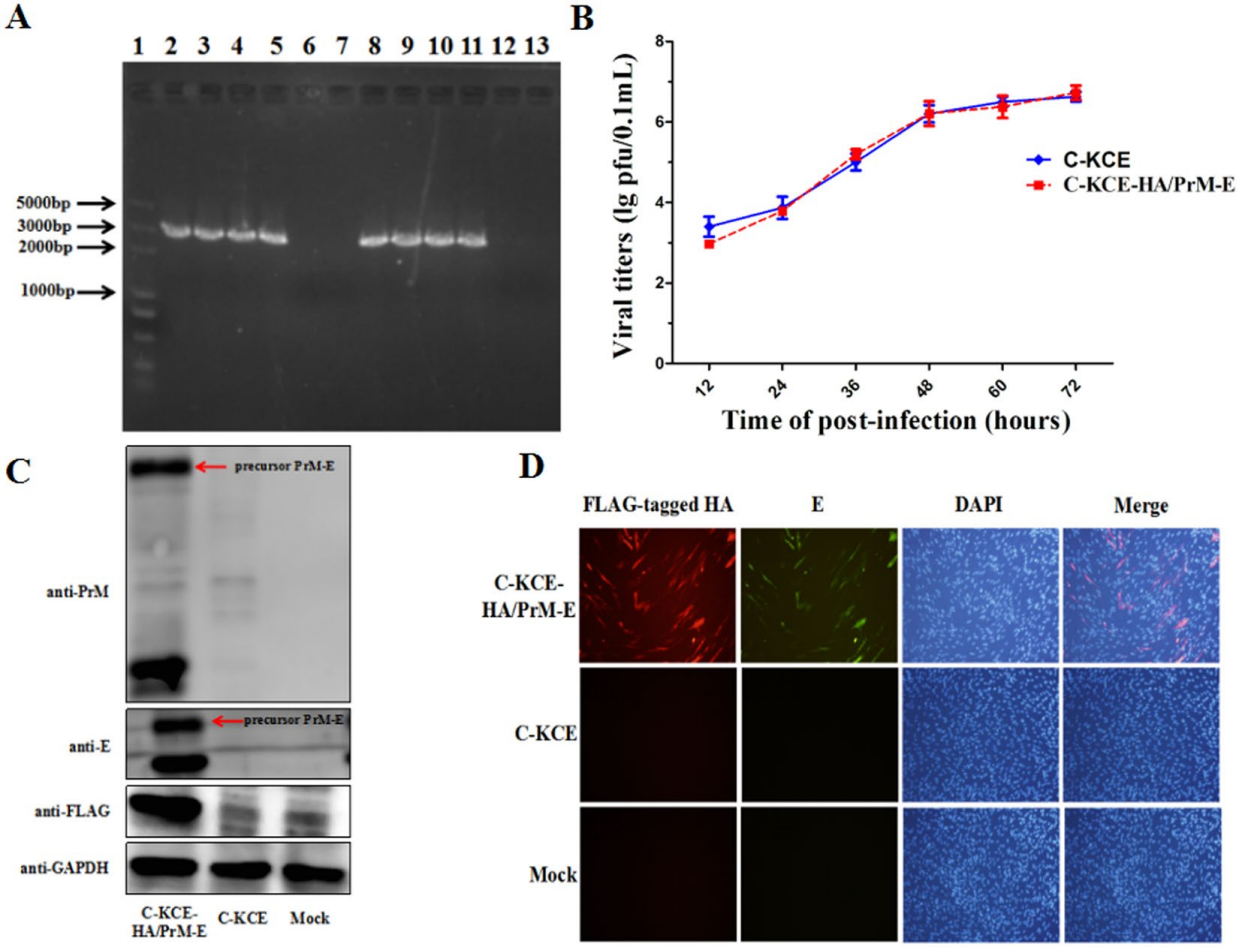
Characterization of the recombinant C-KCE-HA/PrM-E. (**A**) Verification of HA and PrM-E insertion in C-KCE by PCR. The marker used was DL2000plus. Lane 1 is the DL2000plus marker. The products in lane 2, 3, 4, and 5 was amplified from samples with primer pairs JDPrM-E-F/JDPrM-E-R, and lane 6 and 7 are negative C-KCE and the H_2_O control. The products in lane 8, 9, 10, and 11 was amplified from samples with primer pairs JDHA-F/JDHA-R, and lane 12 and 13 are negative C-KCE and the H_2_O control. Full-length gel is presented in the Supplementary Figure 1. (**B**) Multiplestep growth curves of C-KCE-HA/PrM-E and C-KCE in CEF cells. (**C**) Detection of HA, PrM, and E proteins expressions in C-KCE-HA/PrM-E-infected CEF cells by western blot. The precursor PrM-E indicated by the red arrowhead. (**D**) Confirmation of the expression of HA and E protein in C-KCE-HA/PrM-E-infected CEF cells by immunofluorescence. CEF cells infected with C-KCE or mock-infected CEF cells were used as controls. Full-length blots are included in the Supplementary 2.

Finally, the insertion regions of recombinant virus were screened by polymerase chain reaction (PCR) amplification, and the PCR products were further confirmed by sequencing. As shown in Fig. 2A, the positive control PCR bands were observed in all the samples except negative C-KCE and the H_2_O control. In addition, no mutations or deletions were detected in the targets region examined by nucleotide sequence analysis. Taken together, these results indicate that the CRISPR/Cas9 system is a powerful tool for obtaining recombinant C-KCE-HA/PrM-E.

### Biological stability and characterization of the recombinants C-KCE-HA/PrM-E

To investigate the genetic stability of C-KCE-HA/PrM-E, the virus was sequentially grown on CEF cells for 20 passages. The viral DNA was extracted and analyzed after each passage using HA-specific and PrM-E-specific PCR. Both HA and PrM-E genes could be detected by PCR amplification (data not shown).

We also tested whether the HA and PrM insertion affected the *in vitro* replication of C-KCE. Interestingly, both C-KCE-HA/PrM-E and C-KCE were able to replicate efficiently in CEF cells, and the multicycle growth kinetics of C-KCE-HA/PrM-E were similar to that of the parental C-KCE (Fig. 2B), indicating that the direct effects of replication of the C-KCE vaccine strain were obscure.

### Expression of the HA, PrM, and E proteins in CEF cells infected with the recombinant C-KCE-HA/PrM-E viruses

Expression of FLAG-tagged HA, PrM, and E proteins in CEF cells infected with C-KCE-HA/PrM-E were examined by western blot and immunofluorescence assay (IFA). As expected, the bands corresponding to the molecular masses of HA (63 kDa), PrM (20 kDa), and E (55 kDa) were clearly visible in the extract of the C-KCE-HA/PrM-E-infected CEF cells (Fig. 2C), thereby demonstrating that HA, PrM, and E were efficiently expressed. Meanwhile, a protein band of approximately 75 kDa (molecular mass of the precursor PrM-E) was also observed (Fig. 2C), suggesting that the cleavage was incomplete. Conversely, HA, PrM, and E were not detected in CEF cells that had been infected with C-KCE empty vector or were mock (PBS)-infected.

We also used IFA to further evaluate the expression of FLAG-tagged HA and E proteins in CEF cells infected with recombinant C-KCE-HA/PrM-E. C-KCE-HA/PrM-E-infected CEF cells were incubated with a mixture of anti-FLAG and anti-E antibodies, both red and green (Fig. 2D) fluorescence signals were observed by fluorescence microscopy. After merging both fluorescent images, the green and red fluorescence signals co-localized to the same CEF cells (Fig. 2D). However, CEF cells infected with phosphate-buffered saline (PBS) (sham-infected) or C-KCE showed neither red nor green fluorescence signals. These results indicate that the HA, PrM, and E proteins were coexpressed in the recombinant C-KCE-HA/PrM-E -infected CEF cells.

### Virus sustaining in tissues infected with C-KCE-HA/PrM-E, immunogenicity evaluation of C-KCE-HA/PrM-E, and clinical protection against lethal DEV challenge

As shown in Supplementary Fig. 3, C-KCE and C-KCE-HA/PrM-E could be detected in all tested tissues with high average viral loads. No significant difference was detected in C-KCE and C-KCE-HA/PrM-E levels in the tested tissues, suggesting that insertion of HA and the PrM-E gene did not change the replication ability of C-KCE *in vivo*.

To test whether inserting HA and PrM-E could influence the immunogenicity of C-KCE, serum samples were collected weekly for four weeks from all ducks vaccinated with C-KCE-HA/PrM-E, C-KCE, or PBS to screen for the NT antibody, a marker of immunogenicity, against the virulent strian HB/10. The NT antibody titers of the PBS-inoculated groups were lower than 2 log2 and considered negative (Supplementary Fig. 4). In contrast, the NT antibody titers of three ducks exceeded 2^3^ in the C-KCE and C-KCE-HA/PrM-E vaccinated groups at one week pv. The NT antibody titers of four ducks reached 2^4^ at two weeks pv. The titers of the two groups dropped rapidly thereafter, but still remained higher than those of the control group (Supplementary Fig. 4). Although the NT antibody titers primed by C-KCE or C-KCE-HA/PrM-E were low and short-lived, no significant difference was observed, indicating that insertion of HA and the PrM-E gene did not alter the immunogenicity of C-KCE.

**Figure 3:**
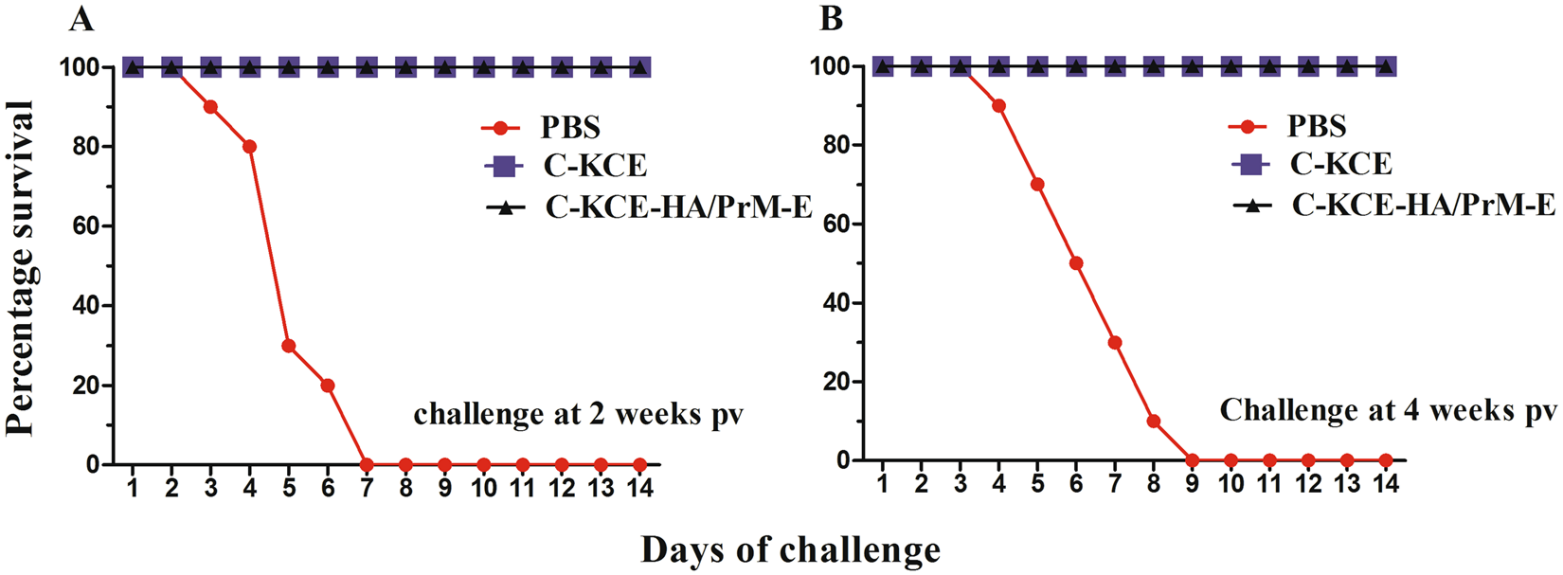
Conferred protection from immunization of ducks with C-KCE-HA/PrM-E against virulent DEV challenge. Ducks were inoculated subcutaneously with 10^5^ PFU of C-KCE-HA/PrM-E, 10^5^ PFU of C-KCE, or PBS (n = 10 per group) as a control, then intramuscularly challenged with 100-fold duck lethal dose (DLD)_50_ HB/10 at 2 weeks (**A**), 4 weeks (**B**) post-vaccination (pv), respectively. Ducks were examined daily for 2 weeks after challenge.

Animal experiments were conducted to test the impact of the inserted foreign genes on the protective efficacy of C-KCE and to evaluate the efficacy of the C-KCE-HA/PrM-E vaccine against a virulent DEV strain HB/10 challenge. All the ducks in group 1 and 2, corresponding to recombinant C-KCE-HA/PrM-E and C-KCE vaccination, respectively, appeared healthy (Fig. 3A and B) without any overt vaccine-induced clinical signs. In comparison, the ducks in the PBS control group exhibited typical clinical signs of the disease starting from 3 d post-challenge (pc), showing listlessness, ruffled feathers, and anorexia, and ultimately succumbed to infection within 9 d (Fig. 3A and B). No difference was observed in the protective efficacy of C-KCE-HA/PrM-E and C-KCE against lethal HB/10 challenge. Thus, the recombinant viruses simultaneously bearing the HA, PrM, and E insertion did not appear to impair the immunogenicity of C-KCE.

### Induction of antibody response in C-KCE-HA/PrM-E-vaccinated ducks

Since neutralization of antibodies is an essential step in mediating protection from virus infection, serum samples were collected at different time points and examined for the presence of neutralization antibodies against both XN/07 and df2.

**Figure 4:**
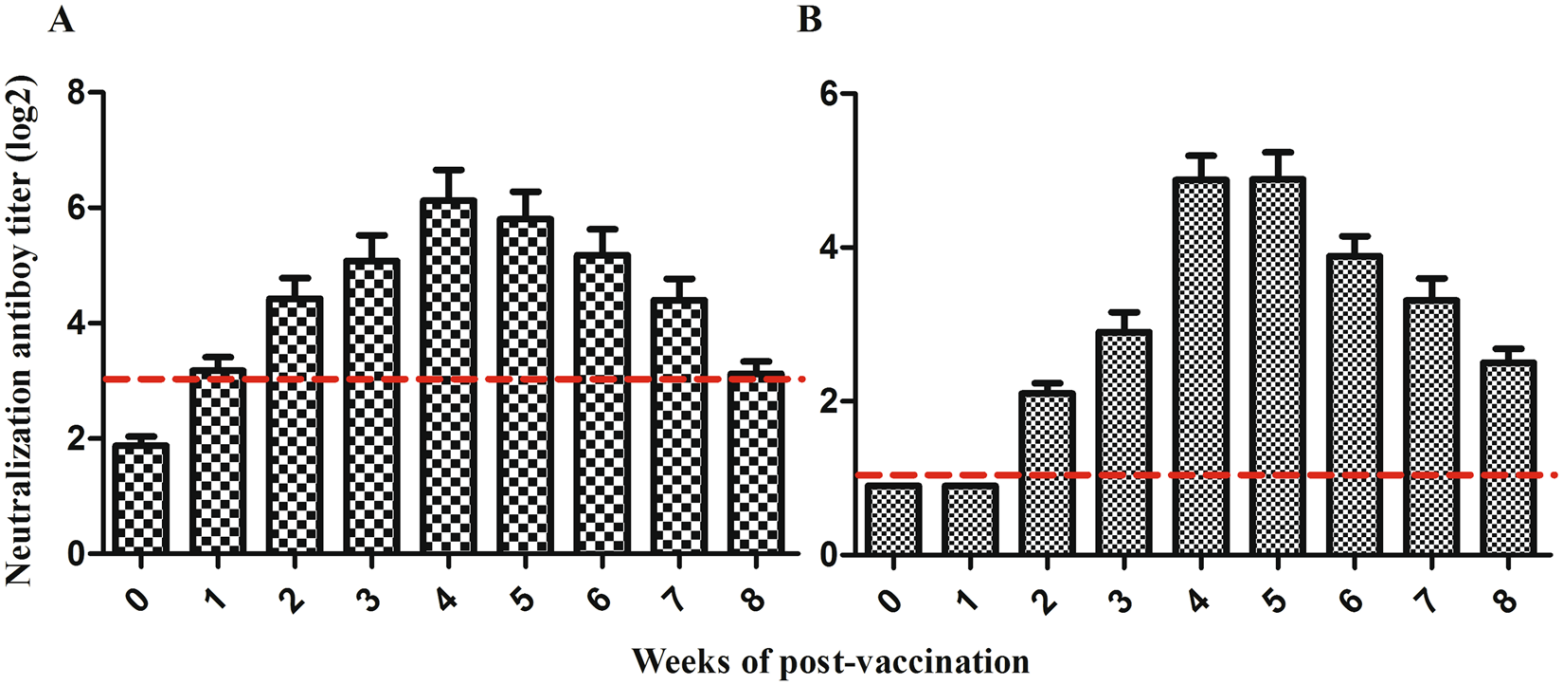
C-KCE-HA/PrM-E viruses prime humoral responses. Ducks were vaccinated with PBS, 10^5^ PFU of C-KCE or C-KCE-HA/PrM-E (n = 10 per group) subcutaneously. Sera were obtained at the indicated time points to detect the neutralization antibodies against XN/07 and df2. (**A**) XN/07-neutralizing antibodies were measured by neutralization assay on MDCK cells. (**B**) df2-neutralizing antibodies were determined by neutralization assay on BHK-21 cells. The dashed line shows the detection limit for a positive response.

No neutralizing antibodies against XN/07 and df2 were detected in the ducks that received PBS and C-KCE vaccinations during the entire experimental period. In agreement with the results of our previous study, the earliest time point of detection of seroconversion in the C-KCE-HA/PrM-E-vaccinated groups was week 2, with mean anti-XN/07 neutralizing antibody titers of 2^3^. Increase in antibody level started at week 3 post-vaccination (pv) with titers of 2^4^, and peaked at week 4 pv with titers of 2^6^. However, the titers gradually declined after 5 weeks pv. Eventually, the neutralizing antibody was only detected in five of the ten ducks, and the levels remained low from 8 weeks pv until the end of our analysis (Fig. 4A). Sera from vaccinated ducks were further evaluated using the HA assay (Supplementary Fig. 5). The trends of HA and neutralizing antibody responses in the C-KCE-HA/PrM-E-inoculated groups were similar, but the levels of HA antibodies were lower than those of neutralizing antibodies at all time points. Similarly, no neutralizing antibodies against df2 were detected at 1 week pv, and limited neutralizing antibodies were detected at 2 weeks pv. The neutralizing antibody titers exceeded 2^3^ at 3 weeks pv, and continued to increase to 2^5^ at 4 weeks pv. However, the peak response level was sustained for only 2 weeks (Fig. 4B). Collectively, these results indicated that ducks vaccinated with C-KCE-HA/PrM-E could elicit humoral immune responses against XN/07 and df2 simultaneously.

### Cellular Response to C-KCE-HA/PrM-E Viral Vaccination

**Figure 5:**
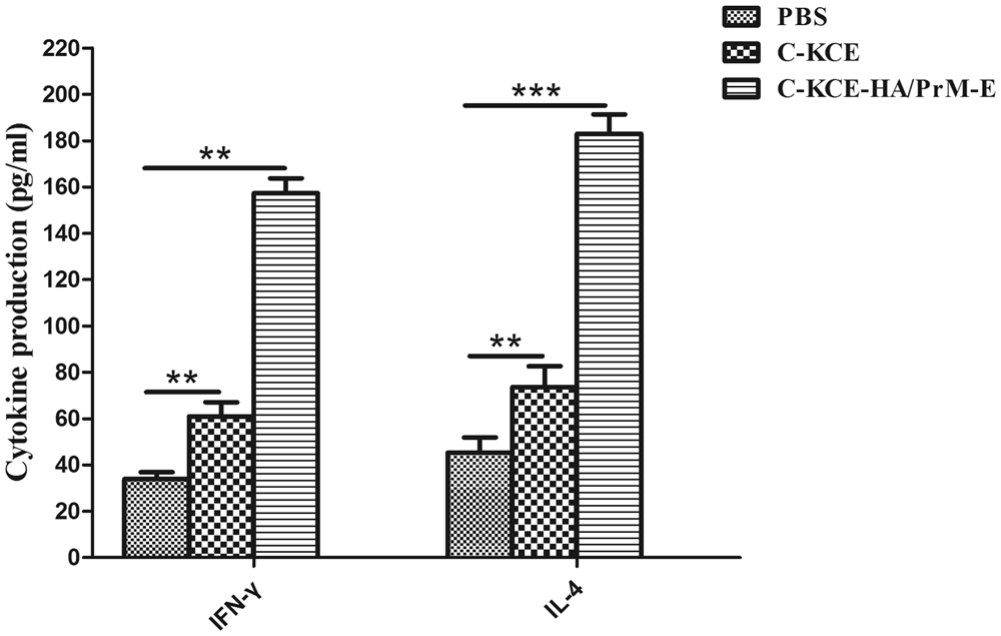
Serum IFN-γ and IL-4 cytokine levels in ducks. Ducks were vaccinated with PBS, 10^5^ PFU of C-KCE or C-KCE-HA/PrM-E (n = 3 per group) subcutaneously. On the day of final immunization (day 14), the serum levels of Th1-type cytokine (IFN-γ) and Th2-type cytokine (IL-4) in ducks were determined by ELISA. Data are shown as the mean ± SD. **P < 0.01, ***P < 0.001.

To determine the serum levels of Th1-type cytokines and Th2-type cytokines, serum levels of IFN-γ and IL-4 were analyzed. As expected, markedly higher levels of IFN-γ and IL-4 were detected in the C-KCE-HA/PrM-E-receiving group compared to the C-KCE and PBS groups (Fig. 5).

**Figure 6:**
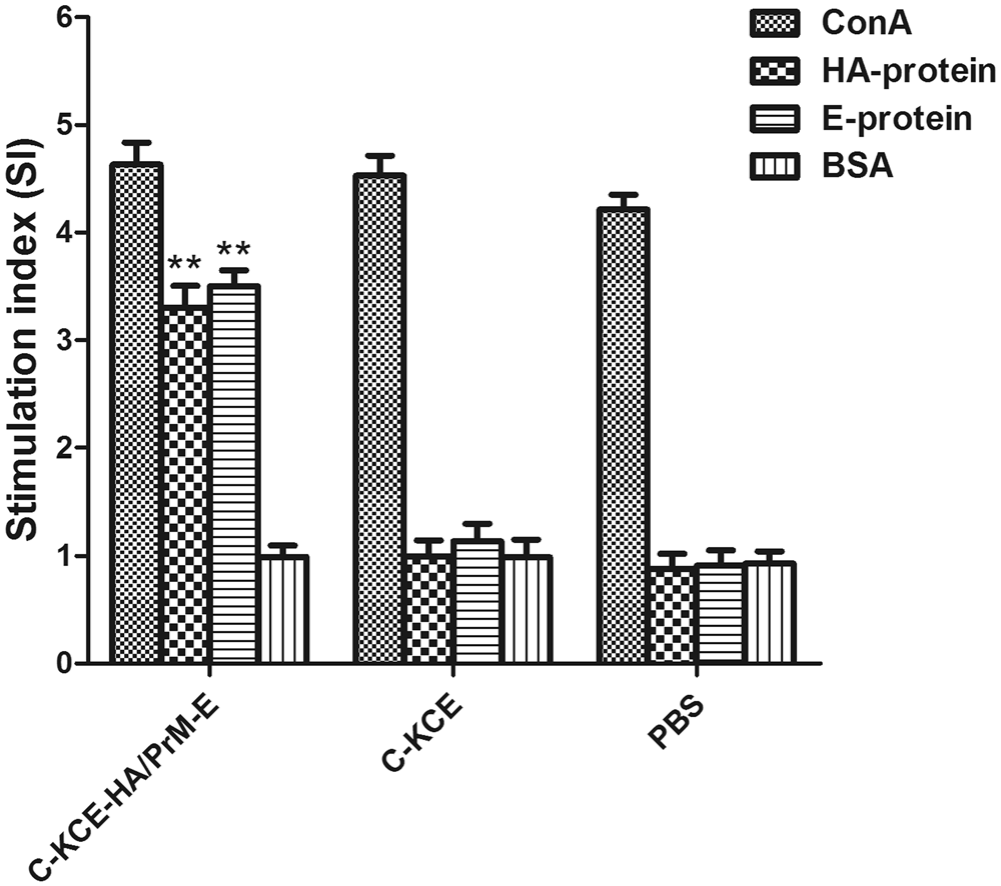
Peripheral blood T-lymphocyte proliferation assay. Ducks were vaccinated with PBS, 10^5^ PFU of C-KCE or C-KCE-HA/PrM-E (n = 3 per group) subcutaneously. On the day of final immunization (day 14), duck peripheral blood lymphocytes were evaluated by MTT assay. Data are shown as the mean ± SD. Statistically significant differences (*P* < 0.01) are indicated by **(compared with C-KCE or PBS group). ConA, Concanavalin A.

Cellular immune response was further evaluated by determining the ability of duck peripheral mononuclear cells to show a proliferative response against purified HA or E protein. As shown in Fig. 6, a significantly enhanced T-cell proliferative response to HA or E protein was clearly observed in the groups immunized with C-KCE-HA/PrM-E when stimulated with purified HA or E protein, whereas the ducks vaccinated with C-KCE or PBS buffer did not respond to the purified HA or E protein. Ducks in all groups responded similarly to stimulation with positive control ConA. These results indicate that C-KCE-HA/PrM-E can elicit cellular immune response in ducks.

### Vaccine efficacy against pathogenic H5N1 and DTMUV isolates challenge in ducks

Given that humoral immune response could be elicited by C-KCE-HA/PrM-E, we next asked whether C-KCE-HA/PrM-E would confer protection against H5N1 and DTMUV challenge. Specifically, the efficacy of C-KCE-HA/PrM-E as a candidate vaccine was evaluated by exposing ducks to H5N1 and DTMUV after vaccination with C-KCE-HA/PrM-E.

**Figure 7:**
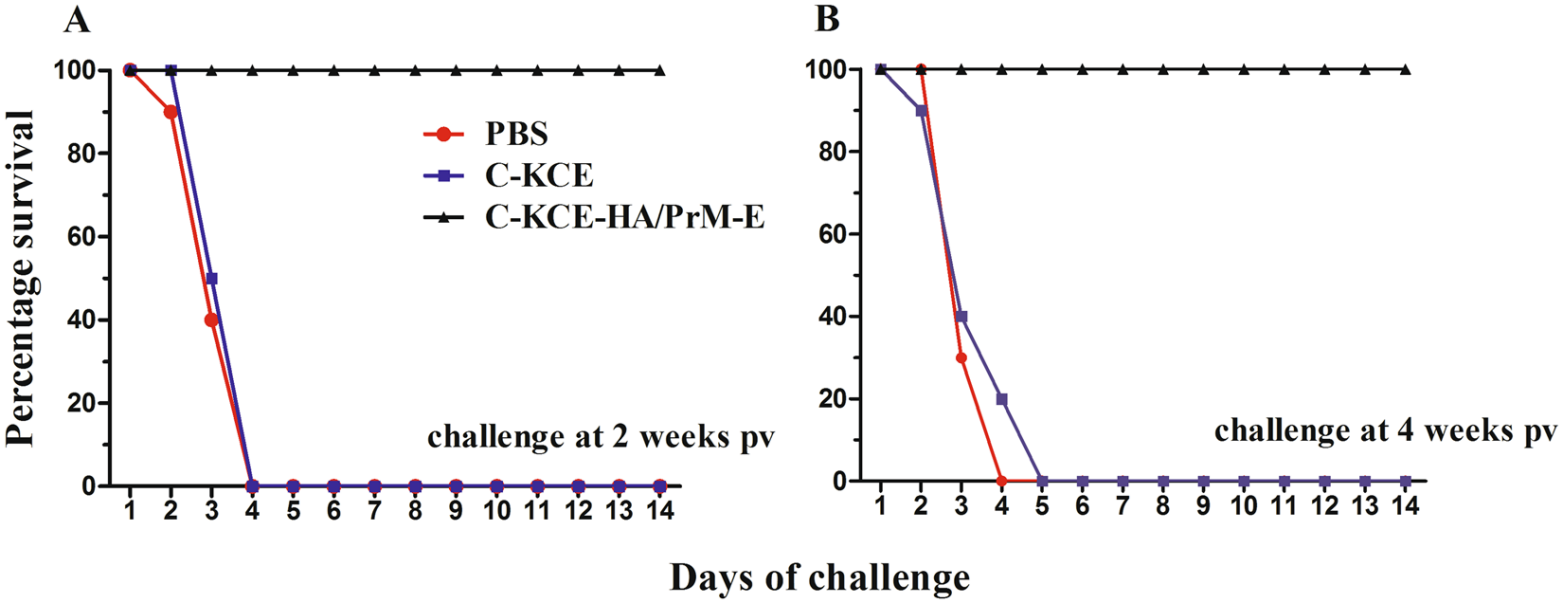
C-KCE-HA/PrM-E protection against HPAIV H5N1 challenge. Ducks were inoculated subcutaneously with 10^5^ PFU of C-KCE-HA/PrM-E, 10^5^ PFU of C-KCE, or PBS (n = 10 per group) as a control, following which they were intramuscularly challenged with 100-fold duck lethal dose (DLD)_50_ XN/07 at 2 weeks (**A**) or 4 weeks (**B**) pv, respectively. The ducks were observed daily for 2 weeks following influenza virus challenge.

After challenge with the XN/07, no clinical signs were observed in the C-KCE-HA/PrM-E-vaccinated ducks, and all ducks survived during the entire 2-week observation period (Fig. 7A and B). Strikingly, the challenge virus XN/07 was not recovered in any of the organs tested at 3 d pc. In contrast, the ducks exposed to C-KCE and PBS showed typical clinical signs of the disease with depression, skin cyanosis, and central nervous system signs from 2–4 d pc and 100% mortality at 5 d pc. As shown in Supplementary Table 1, in the DEV- and PBS-inoculated ducks, the challenged viruses were replicated efficiently in the heart, liver, spleen, lung, kidney, and brain, with high average viral loads ranging from 5.6 to 7.6 log_10_ 50% egg infectious doses (lgEID_50_/g).

Subsequently, we assessed whether C-KCE-HA/PrM-E induced protective immunity against DTMUV challenge. Following infection, all the ducks exposed to C-KCE-HA/PrM-E were completely protected. No virus shedding and clinical signs of this disease were observed. On the contrary, the C-KCE and PBS-vaccinated ducks displayed clinical signs including polydipsia, lethargy, and diarrhea. In addition to clinical signs, df2 infection caused a mild decrease in body weight gain (data not shown). The infected ducks exhibited peak clinical signs between 3–6 d pc. However, the signs gradually decreased thereafter. As shown in Supplementary Table 2, df2 could be detected in all tested tissues including the heart, liver, spleen, lung, kidney, and brain. Notably, the mean viral loads in the organs tested from 4-weeks-old ducks were significantly higher than those of 6-weeks-old infected ducks, which is consistent with a previous report^8^. Taken together, these data demonstrate that C-KCE-HA/PrM-E immunization offered significant protection against H5N1 as well as DTMUV challenge.

## Discussion

Some of the most serious threats facing the duck industry include several infectious pathogens, particularly DEV, HPAIV H5N1, and DTMUV. Currently, vaccines remain the most cost-effective strategy to control these pathogens. However, the available oil adjuvant inactivated vaccines against H5N1 and DTMUV are generally poorly immunogenic in ducks, requiring high antigen doses and multiple immunizations in some cases^29–31^. Moreover, these vaccines are associated with severe adverse reactions in ducks, including local inflammation and “egg drop”. On the other hand, the licensed live vaccines may have certain associated safety concerns^4, 32–34^. Therefore, alternative vaccination strategies are necessary for the prevention and control of H5N1 and DTMUV infections. The present study describes the development of live, subcutaneously delivered C-KCE-vectored vaccines harboring HA, PrM, as well as E, using a CRISPR/Cas9-mediated gene editing strategy, and their examination for genetic stability, safety, immunogenicity, and protective efficacy against virulent DEV, H5N1, and DTMUV challenges in ducks.

Viral vectors have been widely explored for vaccine development. C-KCE, a live attenuated virus vaccine, is a promising vaccine vector against avian pathogens since C-KCE vectors have been shown to be effective at inducing robust immune responses against exogenous antigens. To date, DEV has been widely applied for delivering protective antigens of avian pathogens including HPAIV H5N1, AIV H9N2, DTMUV, as well as duck hepatitis A virus^12, 14, 35, 36^. It is noteworthy that DEV has also been explored as a replicating candidate vaccine vector in chickens to confer protection against the avian infectious bronchitis virus infection and HPAIV H5N1^15, 37^, which has greatly extended the application of DEV. To the best of our knowledge, DEV has not been evaluated as a vaccine vector simultaneously against H5N1 and DTMUV to date. To extend our previous work, we generated a C-KCE-HA/PrM-E construct containing HA, PrM, as well as E. Transfection with recombinant C-KCE-HA/PrM-E had no adverse effects on C-KCE replication *in vitro* or on immunogenicity of the parental C-KCE *in vivo*. Considering that C-KCE-HA/PrM-E harbored both the PrM and E genes, it is not surprising that neutralizing antibodies effective against both H5N1 and DTMUV were elicited. Furthermore, ducks that received a single dose of C-KCE-HA/PrM-E were conferred complete protection against virulent H5N1, DTMUV, and DEV challenges. In addition to efficacy of protection, the clearance of challenge virus in vaccinated ducks is an additional important criterion to evaluate the efficacy of a vaccine^38^. Notably, C-KCE-HA/PrM-E treated ducks were able to rapidly clear the challenged viruses. Thus, the novel C-KCE vector harboring foreign genes shows promise as an effective plaform for development of a multivalent vaccine.

Unlike DEV and DTMUV, H5N1 readily undergoes antigenic drift while circulating in birds. Thus, an update of the H5N1 vaccine strains for preventing infections should be frequently implemented based on viral epidemiology dates. A vaccine that can provide broad protection against homologous and heterologous H5N1 would be ideal. Previously, we have used C-KCE as a vector to deliver the HA gene of H5N1^13^. Ducks that received this vaccine were provided solid protection against challenge by homologous and heterologous H5N1 virus infection. Unfortunately, we did not conduct any tests to analyze whether C-KCE-HA/PrM-E could afford protective immunity against heterologous H5N1. Further study will be required to address this question.

To the best of our knowledge, this is the first study to demonstrate effective use of the CRISPR/Cas9 system to edit the DEV viral genome. CCompared with traditional technologies for modification of DEV, such as bacterial artificial chromosome and the fosmid system, the novel CRISPR/Cas9 approach is more convenient and efficient. The DSBs then trigger DNA repair by at least two distinct mechanisms: NHEJ and HDR^39, 40^. Since NHEJ is error-prone and introduces unpredictable patterns of deletions and insertions, this method is suitable for introducing small random mutations^41, 42^. However, NHEJ does not enable precise genetic modifications by HDR-mediated insertion of an exogenous DNA fragment. HDR is less frequent than NHEJ and only occurs during S and G2 phases^18, 41, 43^, whereas NHEJ occurs throughout the cell cycle^44^. Previous studies have demonstrated that SCR7, an NHEJ inhibitor, enhances the frequency of HDR^23, 45, 46^. By using the CRISPR/Cas9 system combined with SCR7, the recombinant C-KCE-HA/PrM-E strain can be made in less than three weeks. Notably, we provide here the first evidence that HA, PrM, and E, which occur in two different locations on the same DEV genome, could be simultaneously modified with high efficiency and without off-target effects. Importantly, this approach can be used to develop a polyvalent live attenuated vaccine as well to facilitate the study of DEV pathogenesis.

In summary, we have demonstrated here that the CRISPR/Cas9 system is a versatile and powerful technology for targeted engineering of DEV. Our results show that C-KCE-HA/PrM-E generated in the present study could be used as a trivalent vaccine to prevent H5N1, DTMUV, and DEV infection in ducks. Application of this CRISPR/Cas9 system platform to develop a series of vaccines based on C-KCE is expected to be greatly beneficial for combating pathogens in poultry.

## Materials and Methods

### Ethics statements

All of the animal experiments were approved by the Research Ethics Committee, Huazhong Agricultural University, Hubei, China (HZAUMO2015-0015). All the animal experiments were carried out in accordance with the recommendations in the Guide for the Care and Use of Laboratory Animals from Research Ethics Committee, Huazhong Agricultural University, Hubei, China.

### Virus strains

The attenuated DEV C-KCE vaccine strain, obtained from the China Institute of Veterinary Drugs Control, was propagated and titrated in primary CEF cells in Dulbecco’s Modified Eagle’s Medium (DMEM) supplemented with 10% heat-inactivated fetal bovine serum (FBS) and antibiotics at 37 °C under a 5% CO2 atmosphere. A virulent DEV strain (designated as HB/10) isolated from Hubei Province was propagated in 11 d old embryonated duck eggs. AIV H5N1 A/duck/Hubei/xn/2007 (H5N1; designated as XN/07) (GenBank accession number of HA: AHI43271.1) was propagated in the allantoic cavities of 9 d old specific pathogen-free (SPF) embryonated chicken eggs and stored at −80 °C. A virulent DTMUV strain, which adapted to BHK21 through serial passage (GenBank ID: KJ489355) (designated as df2), was also isolated from Hubei province.

### CRISPR/Cas9-mediated DEV cloning

The sequences containing the targeting regions were submitted to CRISPR Design Tool (http://crispr.mit.edu/, Zhang Feng’s Lab), and the gRNAs with highest scores were chosen. The oligos of the gRNAs were synthesized and cloned into sgRNA/Cas9 cloning vector pX330 (catalog no. 42230; Addgene) following the method stated previously^19^. The sequences of the corresponding gRNA oligos are listed in Table 1.

**Table 1:** The gRNA coding sequences and targets sites.

gRNA ID	Targets sites	gRNA Coding Sequence^a^
gRNA-1	UL27 and UL26 gene junction	GGGTCCAATAACGACCGTCGTGG
gRNA-2	US7 and US8 gene junction	CTGGTTAACTGTATTATGCGCGG
gRNA-3	EGFP	GCTGAAGCACTGCACGCCGTAGG
gRNA-4	ERFP	CGGCCACGAGTTCGAGATCGAGG

^a^Underlined text shows the protospacer adjacent motif (PAM) sequence located on the immediate 3′ termination of the gRNA recognition sequence.

The left arm and right arm for the inter-genic region between the UL27 and UL26 genes homologous recombination was amplified by PCR from C-KCE DNA with the primer pairs UL27-F/UL27-R and UL6-F/UL26-R, whereas an enhanced RFP gene and its cassette was amplified by PCR using pdsRED as the template with primers RFP-F/RFP-R. Left arm-RFP-Right arm donor templates were generated using overlapping PCR using primers UL27-F/UL26-R. The left arm and right arm for inter-genic region between the US7 and US8 genes homologous recombination was amplified by PCR from C-KCE DNA with primer pairs US7-F/US7-R and US8-F/US8-R, whereas an enhanced GFP gene and its cassette was amplified by PCR using pEGFP-N1 as the template with primers GFP-F/GFP-R. Left arm-GFP-Right arm donor templates were generated using overlapping PCR with the primers US7-F/US8-R. The left arm and right arm for RFP region homologous recombination was amplified by PCR from C-KCE-RFP/GFP DNA with primer pairs HA-1F/HA-1R and HA-3F/HA-3R. The (HA) gene was represented as A/duck/Hubei/xn/2007 with FLAG-tagged at the C-terminal synthesized with several mutations at the polybasic cleavage site, as previously described. Subsequently, HA was amplified by PCR using the primers HA-2F/HA-2R. Left arm-HA-Right arm donor templates were generated using overlapping PCR with the primers HA-1F/HA-3R. The left arm and right arm for GFP region homologous recombination was amplified by PCR from C-KCE-RFP/GFP DNA with primer pairs PrM-E-1F/PrM-E-1R and PrM-E-3F/PrM-E-3R, whereas a PrM and E gene was amplified by PCR using cDNA from DTMUV df2 as the template with primers PrM-E-2F/PrM-E-2R. Left arm-HA-Right arm donor templates were generated using overlapping PCR with the primers PrM-E-1F/PrM-E-3R. The sequences of Left arm-RFP-Right arm, Left arm-GFP-Right arm, Left arm-HA-Right arm, and Left arm-PrM-E-Right arm fragments were confirmed by Sanger sequencing, respectively. All primer pairs used in the present study are listed in Table 2.

**Table 2:** Primers used for generating C-KCE-HA/PrM-E, identification of the C-KCE-HA/PrM-E, and determination the DTMUV load.

Primer ID	Sequence (5′ → 3′)^a^
UL27-F	AGAAATATTACCCCTTCAAGTATATACT
UL27-R	**CCACACCCCACG**AAAAATGGAAACACGGGGGA
RFP-F	**GTTTCCATTTTT**ATAATACAGTTAACCA
RFP-R	**CGGTTTCTTCGT**GCGGCCGCTAGGGATA
UL26-F	**CCTAGCGGCCGC**ACGAAGAAACCGCCCAACG
UL26-R	TCGGGGCCAGTGGTGCCTACTGGA
US7-F	TACCGGATGTGTTTAAAACGACAGTTAT
US7-R	**TATTAATAACTA**ATAATACAGTTAACCA
GFP-F	**TAACTGTATTAT**TAGTTATTAATAGTAATCAA
GFP-R	**CAAACTCCGCGC**CGCTTACAATTTACGCCTTA
US8-F	**AAATTGTAAGCG**GCGCGGAGTTTGGAGT
US8-R	AGGGCATGTGTTGTCAACTATAGGCTTC
HA-1F	CTTCCCTCCCCCGTGTGGTGCTTT
HA-1R	**CACTATTTTCTC**CATGGTGGCCTAAGGA
HA-2F	**TAGGCCACCATG**GAGAAAATAGTGCTTC
HA-2R	**GCCCTGAGGCTA**CTTATCGTCGTCATCC
HA-3F	**GACGACGATAAG**TAGCCTCAGGGCTAGA
HA-3R	TGCGGTCGTGGCTATAGGAGGAAC
PrM-E-1F	TATGATTGACTGTTTGCCTTTCAT
PrM-E-1R	**GTCGAGCATCTG**CATGGTGGCGACCGGT
PrM-E-2F	**GTCGCCACCATG**CAGATGCTCGACGGAC
PrM-E-2R	**GCGGCCGCTTTA**GGCATTGACATTTACT
PrM-E-3F	**AATGTCAATGCC**TAAAGCGGCCGCGACT
PrM-E-3R	GACGAATCATTTATACGTGCGTGT
JDHA-F	CCAAAGCTGTTGCGTCTC
JDHA-R	AACACGTACAGTTTCGTT
JDPrM-E-F	TTTGTACATGAGGTAATA
JDPrM-E-R	CGTACCAATTGTTGAGGT
NS1-F	GACACGGGGTGCTCAATCGACT
NS1-R	AGCCATGACCTTTGATTTGATC
QT-NS1-F	GGACAACAACAGCGAGTGGGAA
QT-NS1-R	CATGTCGTCTCCGTGAGCAGTT
DEV-F	AAATCTGCTTGCCGGGGATACC
DEV-R	TGCGGCGAAGAGGCGCAAGCTA
QT-DEV-F	TTTTCCTCCTCCTCGCTGAG
QT-DEV-R	ACTTCTGCAAACCCGGCC

^a^The overlap sequences are marked in bold.

### CRISPR/Cas9-mediated homologous recombination and fluorescence-activated cell sorting

CEF cells were plated in a well of a six well dish the day before transfection. The gRNAs were cotransfected with Cas9 and the donor fragments into CEF cells. At 12 h after transfection, the CEF cells were maintained in DMEM supplied with 1 μM SCR7 (DNA ligase IV, Excess Bioscience; no. M60082-2s). After 24 h, the CEF cells were infected with 10 MOI of C-KCE or C-KCE-GFP/RFP. The infected CEF cells were harvested at the indicated time points, and then frozen at −80 °C for screening the target recombinant virus. The fluorescence-activated cell sorting was conducted on a MoFlo XDP sorter (Beckman Coulter) according to the manufacturer’s protocol. CEF cells were infected with the first generation recombinant C-KCE-HA/PrM-E at an MOI of 0.1. The single cells were double positive for both GFP and RFP signals sorted into a 24 well plate pre-seeded with CEF cells.

### Verification of mutant recombinant virus C-KCE-HA/PrM-E

CEF cells were plated in a well of a six well dish on the day of virus infection. CEF cells were infected with purified C-KCE-HA/PrM-E. Infected CEF cells were harvested after 48 h of infection. C-KCE-HA/PrM-E DNA was extracted with an EasyPure Viral DNA/RNA Kit (Qiagen, Valencia, CA) following the manufacturer’s protocol. To verify insertion of the HA gene, a DNA fragment spanning the gene junction region between UL27 and UL26 was amplified by PCR with the primers JDHA-F/JDHA-R. To verify insertion of the PrM-E gene, a DNA fragment spanning the gene junction region between the US7 and US8 was amplified by PCR with the primers JDPrM-E-F/JDPrM-E-R (Table 2). The identities of the PCR products were then cloned into T-vectors for sequencing to determine the mutations or deletions.

### Confirmation of the expressions of the HA, PrM, and E proteins in CEF cells infected with the C-KCE-HA/PrM-E

Expressions of the HA, PrM, and E proteins from CEF cells infected with the recombinant C-KCE-HA/PrM-E virus were evaluated by IFA and western blot assay with monoclonal antibody (MAb) against FLAG tag (Santa Cruz Biotechnology, CA, USA), polyclonal antibody (PAb) against PrM (previously prepared in our laboratory), PAb against E (previously prepared in our laboratory), or MAb against GAPDH (Santa Cruz Biotechnology, CA, USA) for the control. Details of the procedure have been described previously^47^.

### Stability and growth properties of the recombinant C-KCE-HA/PrM-E virus

To examine the genetic stability of the recombinant C-KCE-HA/PrM-E virus, the virus was sequentially grown on CEF cells for 20 passages, and viral DNA was extracted and analyzed after each passage by PCR with the primers JDHA-F/JDHA-R and JDPrM-E-F/JDPrM-E-R (Table 2). To compare the growth of C-KCE and C-KCE-HA/PrM-E, a multicycle growth kinetic assay was conducted as previously described^48^.

### Animal experiments

SPF ducks were purchased from the Harbin Veterinary Research Institute, China. A total of 263 2-weeks old SPF ducks were enrolled in the present study. Animal experiments were conducted to test the immunogenicity and protective efficacy of the C-KCE-HA/PrM-E vaccine against DEV, HPAIV H5N1, and DTMUV.

For examining immunogenicity of C-KCE-HA/PrM-E against DEV, we subcutaneously inoculated three groups of ducks (five per group) with 10^5^ PFU of C-KCE-HA/PrM-E, C-KCE, or PBS as control. At 0, 1, 2, 3, and 4 weeks post-vaccination (pv), serum samples were obtained weekly from all ducks to screen for neutralizing (NT) antibodies against DEV.

To determine the number of viruses sustaining in organs infected with C-KCE-HA/PrM-E or C-KCE, we subcutaneously inoculated two groups of ducks (three per group) with 10^5^ PFU of C-KCE-HA/PrM-E or C-KCE. All ducks in the two groups were humanely euthanized on day 2 post-challenge (pc), and their organs, including heart, liver, spleen, lung, spleen, brain, duodenum, rectum, thymus and bursa were obtained to determine virus titers using a one-step real-time TaqMan RT-PCR assay.

For clinical protection of C-KCE-HA/PrM-E against virulent DEV challenge, three groups of ducks (twenty per group) were inoculated subcutaneously with 10^5^ plaque-forming units (PFU) (a recommended dose for the DEV vaccine) of C-KCE or C-KCE-HA/PrM-E, whereas naive control ducks were inoculated with PBS. Each group of ducks was then randomly subdivided into two groups. The ducks were challenged with a 100-fold 50% duck lethal dose (DLD_50_) of HB/10 by intramuscular injection either at 2 weeks or 4 weeks pv. Ducks were observed daily for the signs of disease and death for 2 weeks pc.

To evaluate the serological responses against HPAIV XN/07 and DTMUV df2 stimulated by immunized C-KCE-HA/PrM-E in ducks, ducks were randomly divided into three groups (ten per group), and each received one immunization subcutaneously with 10^5^ PFU of C-KCE-HA, C-KCE, or PBS as a negative control. For evaluating the serological responses against HPAIV XN/07 and DTMUV df2, serum samples were collected weekly for 8 weeks from all the groups to monitor the neutralization antibodies.

To test the ability of the trivalent C-KCE-HA/PrM-E to provide protection against HPAIV XN/07, 60 ducks were randomly divided into six groups (thirteen per group). Two groups of ducks were inoculated subcutaneously with 10^5^ PFU of C-KCE-HA/PrM-E, and the remaining four groups were inoculated with 10^5^ PFU of C-KCE or PBS as a negative control. Each treatment duck was then intramuscularly challenged with a 100-fold DLD_50_ of XN/07 at 2 weeks and 4 weeks pv. Three ducks in each group were humanely sacrificed on day 3 pc, and their organs, including heart, liver, spleen, lung, kidney, and brain, were collected for virus titration. Ducks were monitored daily for signs of disease and death for 2 weeks pc. The animal experimental design for evaluating the clinical protection of C-KCE-HA/PrM-E against DTMUV df2 was the same as that used for XN/07 described above, and viral loads in the heart, liver, spleen, lung, kidney, and brain of ducks in each group were examined by the quantitative Reverse transcription polymerase chain reaction (RT-PCR).

### Lymphocyte proliferation assay, detection of IFN-γ and IL-4, serologic tests and virus titration

To detect cellular responses to immunization with C-KCE-HA/PrM-E in ducks, 14 days after the final immunization, blood samples from 3 ducks in each group were collected for lymphocyte proliferation assay. The peripheral blood lymphocyte proliferation assay was performed using a modified MTT method as described previously^49, 50^. To determine the serum levels of Th1-type cytokines and Th2-type cytokines, serum levels of IFN-γ and IL-4 were analyzed using commercial duck IFN-γ and IL-4 sandwich ELISA kits following the manufacturer’s instruction. NT antibody against HB/10 was tested in DEF as described previously^13^. Serum samples were obtained to monitor HA-specific antibodies via HI assays and neutralizing antibodies against XN/07 were determined in MDCK cells using 1% chicken red blood cells as described previously^51^. Detection of neutralizing antibodies against DTMDV df2 was performed in BHK-21 cells following the method described previously^36^. For each organ, a 1 g sample was collected and mixed into 1.0 mL PBS with 200 μg/mL penicillin and 200 μg/mL streptomycin, and then homogenized and clarified by centrifugation. XN/07 titration was conducted in 9 d old SPF embryonated chicken eggs by EID_50_ assay, whereas DTMUV df2 was examined by quantitative RT-PCR assay as previously described with some modification^8^. Briefly, the primers NS1-F/NS1-R for viral NS1 gene derived from DTMUV were designed to amplify the fragment as a standard template (Table 2). Subsequently, the fragment was cloned into pGEM-T Easy vector following the manufacturer’s instructions. The primers QT-NS1-F/QT-NS1-R (Table 2) were designed for quantitative RT-PCR. C-KCE and C-KCE-HA/PrM-E were determined using a one-step real-time TaqMan RT-PCR assay. Briefly, the primers DEV-F/DEV-R for viral genes derived from C-KCE were designed to amplify the fragment as a standard template (Table 2). Subsequently, the fragment was cloned into the pGEM-T Easy vector following the manufacturer’s instructions. The primers QT-DEV-F/QT-DEV-R (Table 2), and probes (5′-FAM-CCCTGGGTACAAGCG-MGB-3′) were designed for quantitative RT-PCR. One-step, real-time TaqMan RT-PCR assays were carried out in an Applied Biosystems 7500 Fast real-time PCR system (Life Technologies, Carlsbad, CA, USA).

### Statistical analysis

All experiments were reproducible and performed in triplicates. Statistical analyses were conducted by a one-way ANOVA test to compare the data of the difference groups using GraphPad Prism version 5.0 (GraphPad Software, La Jolla, CA, USA). p-values of <0.05 were considered statistically significant.

### Laboratory facility

All experiments involving live viruses were performed in a biosafety level 3 (BSL3) facility in accordance with the institutional biosafety manual. Animals were housed in negative-pressure isolators with HEPA filters in the BSL3 facility.

## Electronic supplementary material

CRISPR Supplementary materials
