# Supplementary material for: Construction of a highly efficient CRISPR/Cas9-mediated duck enteritis virus-based vaccine against H5N1 avian influenza virus and duck Tembusu virus infection
Source: Sci Rep. 2017 May 3;7:1478. doi: 10.1038/s41598-017-01554-1 (PMC5431151; doi:10.1038/s41598-017-01554-1)
Supplement: Supplementary file 1 — CRISPR Supplementary materials [file 41598_2017_1554_MOESM1_ESM.pdf]

Construction of a highly efficient CRISPR/Cas9-mediated duck enteritis virus-based vaccine against H5N1 avian influenza virus and duck Tembusu virus infection

Zhong Zou; Kun Huang; Yanmin Wei; Huanchun Chen; Ziduo Liu; Meilin Jin

Supplementary materials

Supplementary Figure 1. Verification of HA and PrM-E insertion in C-KCE by PCR.

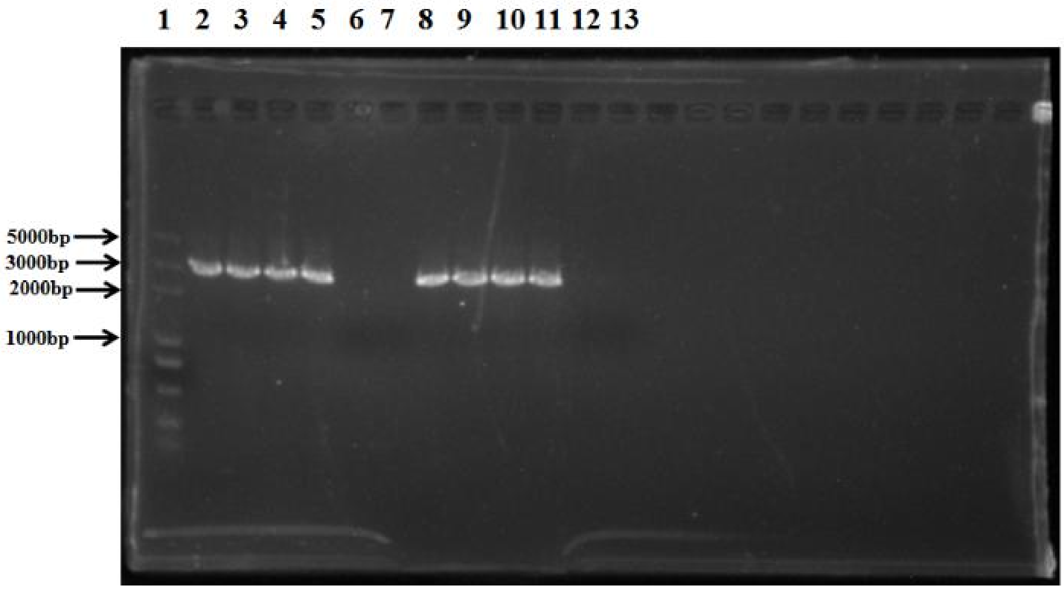

Supplementary Figure 2. Detection of HA, PrM, and E proteins expressions in C-KCE-HA/PrM-E-infected CEF cells by western blot. The precursor PrM-E, PrM, and E indicated by the red arrowhead. A and B are the different exposures.

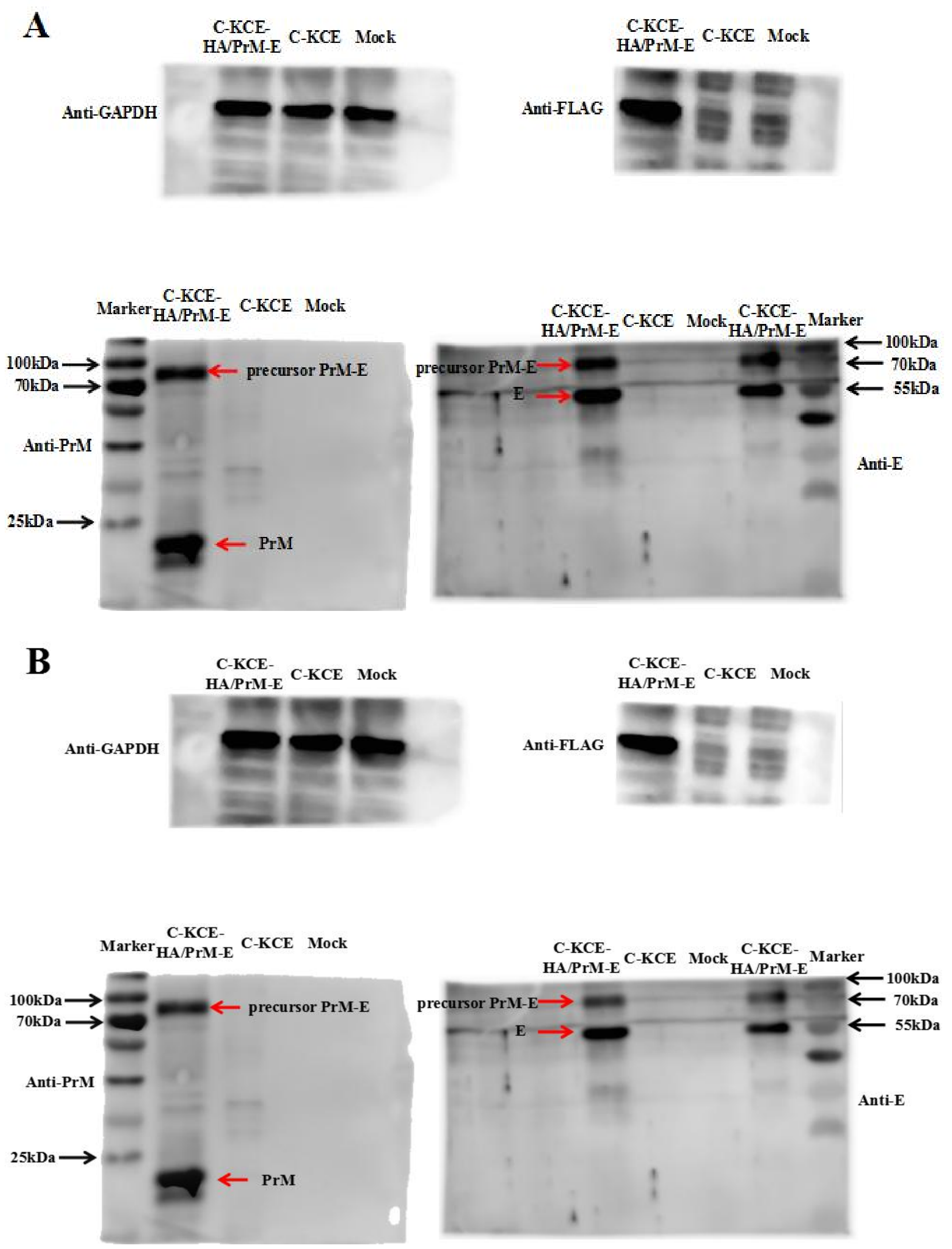

Supplementary Figure 3. Viral load in organs infected with C-KCE-HA/PrM-E or C-KCE. Ducks were vaccinated with 10^5 PFU of C-KCE or C-KCE-HA/PrM-E (n = 3 per group) subcutaneously. All the ducks in the two groups were humanely euthanized on day 2 post-challenge (pc), and their organs were obtained to determine virus titers using a one-step real-time TaqMan RT-PCR assay.

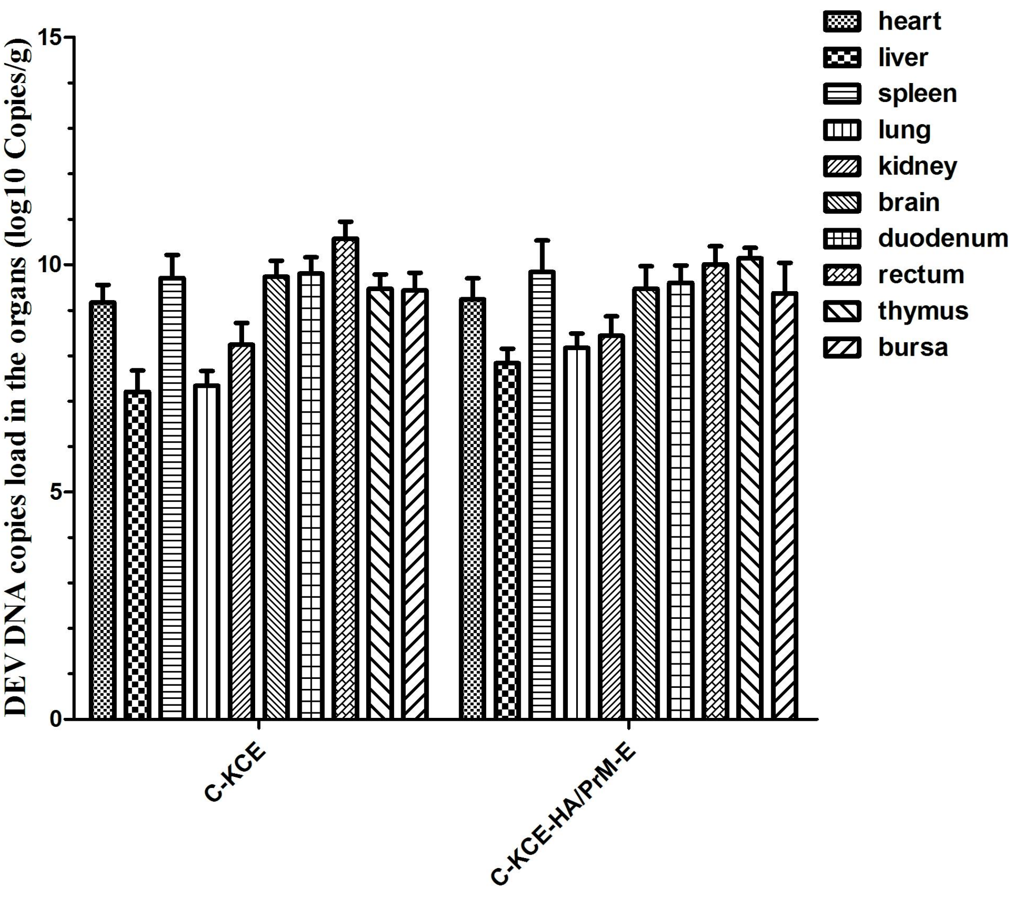

Supplementary Figure 4. Humoral immune response against virulent DEV in ducks vaccinated with C-KCE-HA/PrM-E or its parental strain C-KCE. Groups of 5 ducks were inoculated subcutaneously with 10^5 PFU of C-KCE-HA/PrM-E, C-KCE or with PBS as control. Sera were collected from 0 to 4 weeks to detect NT antibodies against virulent DEV in DEF cells. NT antibody titers for ducks are shown in log2 scale. Dotted lines indicate the thresholds for a positive response. Data are shown as the means ± SD. ** P < 0.01, *** P < 0.001.

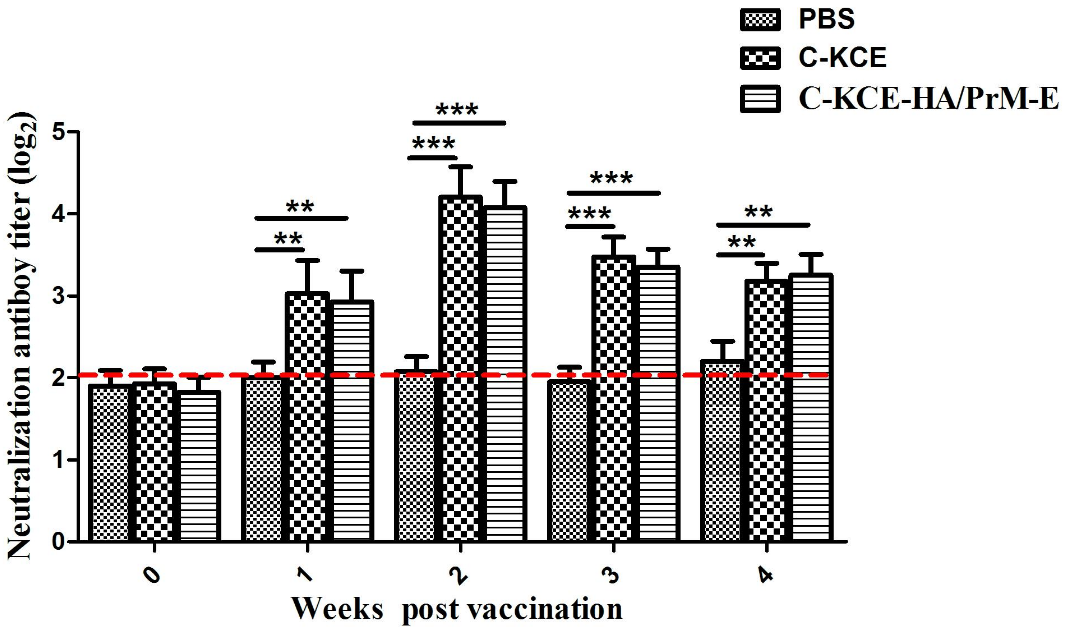

Supplementary Figure 5. HA responses were assessed against the homologous XN/07 virus. Ducks were vaccinated with PBS, 10^5 PFU of C-KCE or C-KCE-HA/PrM-E (n = 10 per group) subcutaneously. Sera were obtained at the indicated time points to detect HA antibodies against XN/07. Dotted lines indicate the thresholds for a positive response.

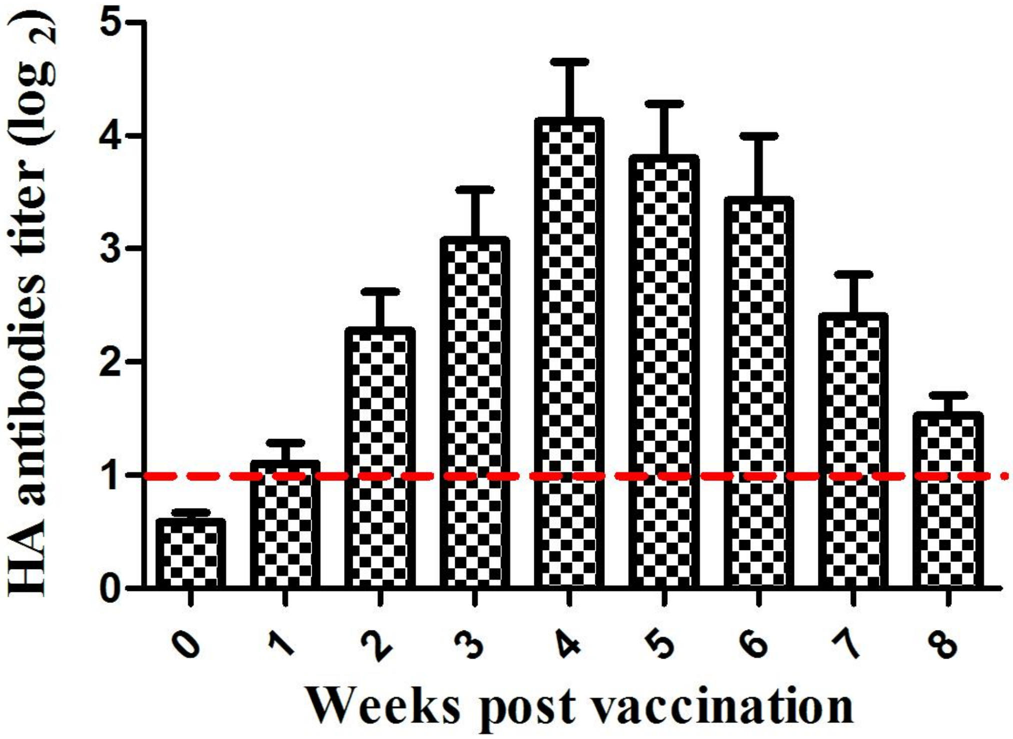

Supplementary Table 1. Replication of challenge virus XN/07 in ducks.

Challenge time pv	vaccine	Virus replication in the organs in the ducks on 3 days pv (mean ± SD, lg10EID$_{50}$/g)					
		Heart	Liver	Spleen	Lung	Kidney	Brain
2 weeks	C-KCE-HA-PrM-E	/	/	/	/	/	/
	C-KCE	6.3± 1.1	7.1±0.5	6.8±0.7	5.8±1.1	6.5±0.9	7.2±0.6
	PBS	6.7± 0.8	6.4±0.6	7.0±0.5	6.8±1.4	6.2±0.8	7.5±0.8
4 weeks	C-KCE-HA-PrM-E	/	/	/	/	/	/
	C-KCE	6.4±0.7	5.6±1.3	6.3 ±1.0	5.4±0.9	7.6±0.4	7.3±0.4
	PBS	6.2±0.5	7.0±0.4	6.8±0.7	6.6±1.2	7.1±0.5	6.8±0.7

Homologous XN/07 replication in the organs of ducks that were vaccinated with C-KCE-HA/PrM-E. Groups of three ducks were inoculated subcutaneously with 10^5 plague-forming units (PFU) of C-KCE-HA, C-KCE, or PBS as a control. Subsequently, the ducks were challenged with homologous (XN/07) intramuscularly at 2 weeks or 4 weeks pv. At day 3 after challenge, the ducks were humanely sacrificed and their organs were collected for virus titration in eggs. Data represent mean titers ± standard errors. The backslash indicates that the challenge virus was not recovered at the corresponding time point.

Supplementary Table 2. Replication of challenge virus df2 in ducks.

Challenge time pv	vaccine	Viral copy load in the organs in the ducks on 3 days pv (mean ± SD, log_{10} per µg of total RNA)					
		Heart	Liver	Spleen	Lung	Kidney	Brain
2 weeks	C-KCE-HA-PrM-E	/	/	/	/	/	/
	C-KCE	5.91 ± 0.54	5.73± 0.28	6.18± 0.42	4.29± 0.47	5.16± 0.33	2.69 ± 0.43
	PBS	6.13 ± 0.22	5.42± 0.37	5.78± 0.33	5.14± 0.26	5.46± 0.26	3.17 ± 0.21
4 weeks	C-KCE-HA-PrM-E	/	/	/	/	/	/
	C-KCE	5.27 ± 0.34	4.82± 0.33	5.16± 0.27	4.52± 0.26	4.27± 0.26	2.26 ± 0.52
	PBS	4.66 ± 0.29	4.43± 0.47	5.45± 0.18	4.19± 0.33	4.66± 0.42	2.53 ± 0.33

DTMUV replication in the organs of the ducks vaccinated with C-KCE-HA/PrM-E . Groups of three ducks were inoculated subcutaneously with 10^5 PFU of C-KCE-HA/PrM-E, C-KCE, or with PBS as the control. Subsequently, the ducks were challenged with df2 intramuscularly at 2 weeks or 4 weeks pv. Ducks were euthanized on day 3 post-challenge, and their organs were harvested for virus titration utilizing the quantitative RT-PCR assay. Data are expressed as means ± standard deviations of log_{10}. The backslash indicates that the challenge virus was not recovered at the corresponding time point.
